# Study on the structural stability of partial cable-stayed bridges with multiple towers and high piers during construction

**DOI:** 10.1371/journal.pone.0310631

**Published:** 2024-12-12

**Authors:** Chenglong Zhu, Lingbo Wang, Yixiang Liu, Lin Kang, Xinjun Guo, Xiaobo Zheng

**Affiliations:** 1 School of Highway, Chang’an University, Shaanxi, Xi’an, China; 2 Key Laboratory of Transport Industry of Bridge Detection Reinforcement Technology, Chang’an University, Shaanxi, Xi’an, China; 3 Engineering Management Department of Guangxi Zhonghe Expressway Co., LTD, Guangxi, China; 4 Hangzhou Institute of Communications Planning Design & Research Co., LTD, Hangzhou, China; 5 Shaanxi Institute of Teacher Development, Shaanxi Normal University, Shaanxi, Xi’an, China; University of Zanjan, ISLAMIC REPUBLIC OF IRAN

## Abstract

To clarify the structural safety and stability of partial cable-stayed bridges with multiple towers and high piers during the construction stage, a finite element analysis model of the entire construction process was established using a five-tower, six-span, partial cable-stayed bridge in Shaanxi Province, China, as the engineering background. Linear elasticity and nonlinear stability analyses were carried out in the following key construction stages: bare tower construction, maximum cantilever construction without cables, maximum cantilever construction with cables, side-span closure, secondary mid-span closure, mid-span closure, and second-phase paving. A sensitivity analysis of the structural parameters (the main tower stiffness, main pier stiffness, and main beam stiffness) was conducted. Furthermore, the impacts of the combination of the reduction of the main pier stiffness with two unfavorable factors (the detachment of the construction hanging basket and asymmetric construction on both sides of the main beam) on the construction safety and stability of the structure were explored. The results show that the stability coefficient of the structure during the entire construction process met the requirements of the corresponding specifications; the linear elastic stability coefficient at each stage was greater than 4.0, and the nonlinear stability coefficient was greater than 2.5. Moreover, the stability coefficient was found to be the smallest at the stage of maximum cantilever construction with cables, which was thus identified as the most unfavorable construction stage. The instability mode at each construction stage was found to be the longitudinal instability of the main pier. The structural parameter sensitivity analysis revealed that the change in the stiffness of the main pier had the greatest impact on structural stability, and the structural safety factor was found to be proportional to the stiffness of the main pier. Furthermore, the effects of multiple factors were found to intersect; under the combination of the reduction of the main pier stiffness with the two unfavorable factors, the stability coefficient of the structure was found to be greatly reduced by more than 15%. Therefore, during the construction of partial cable-stayed bridges with multiple towers and high piers, the stage of maximum cantilever construction with cables should be of primary focus, the maintenance and management of the piers should be strengthened, and the unfavorable factors should be avoided. The results of this study can provide a reference for the construction and design of similar types of bridges.

## 1 Introduction

Partial cable-stayed bridges, also called "low-tower cable-stayed bridges," combine the advantages of economy, beauty, short towers, rigid beams, concentrated cables, and convenient construction, and have attracted widespread attention from engineers around the world [1–3]. The design concept of partial cable-stayed bridges originated from the famous French bridge engineer Jacques Mathivat. As compared with cable-stayed bridges, the main tower height of these composite bridges is shorter, the main beam stiffness is higher, and the stay cables are relatively concentrated [4–7].

The forces on partial cable-stayed bridges are relatively complex; this is especially true for the construction of long-span partial cable-stayed bridges, which often undergo multiple system transformations [5, 8]. During the construction process, the bridges are affected by the initial deformation of the structure, the tension force of the cables, and the shrinkage and creep of the concrete. These factors cause substantial changes in the internal force of the structure, and the most unfavorable loads often appear during construction [9, 10]. For cable-stayed bridges with multiple towers and high piers, stability problems during construction are extremely prominent, and stability analysis is necessary to ensure their safety [11]. Furthermore, structural stability is one of the main issues related to safety and economy, and is of equal importance to issues such as structural strength [12, 13]. Therefore, it is of great significance to carry out stability research, reveal the impacts of relevant factors on structural stability, and ensure the safety and quality of the structure during the entire construction phase [14–17].

Research on the construction technology of partial cable-stayed bridges has previously been carried out. For instance, Pei et al. [18] introduce the construction technology of long-span cable-stayed bridge. This new method is simple and fast, can reduce the investment of auxiliary materials and personnel, and can also shorten the construction period for which cables are needed. Li [19] carried out the structural design verification of the temporary consolidation of piers and beams during the construction of the main beam of a short-tower cable-stayed bridge. They elaborated on the verification method and key points of the temporary pier and beam consolidation structure, thus providing a reference for the design of similar structures. Xiong et al. [20] studied the feasibility of the synchronous construction of the towers and beams of cable-stayed bridges; they demonstrated that the stress state of the main tower after the adjustment of the cable tension was basically consistent with that when the towers were constructed before the beams. Yan et al. [21] studied two construction methods (i.e., the synchronous construction of towers and beams, and initial tower construction followed by beam construction); the results revealed that the axial force of the main beam was basically the same under the two methods. Han et al. [22, 23] studied the influence of the delayed tensioning of the stay cables of a short-tower cable-stayed bridge on the structure, and verified the reasonability of the delayed construction technology of the cable stays. Lu et al. [24, 25] investigated the use of ultra-wide blocks in the construction of short-tower cable-stayed bridges, which they found to have significant lateral effects; as such, the authors asserted that these blocks should be taken seriously during construction. Luo et al. [26] determined the best closure plan for a multi-span, low-tower, cable-stayed bridge based on an existing project to provide a reference for similar bridges. Wang et al. [27] studied the causes of oblique cracks in the web during the construction of a concrete box girder with a wide central cable plane and a short-tower cable-stayed bridge; they found the main causes of cracks to be the temperature field in the box during the curing period, the temperature change of the web surface before and after removing the formwork, and the radial force of the tension of the downward bending steel of the web.

These previous studies primarily focused on the construction technology of partial cable-stayed bridges, and there has been a lack of research on the stability of partial cable-stayed bridges during their construction. In particular, although the safety and stability issues during the construction of such bridges with multiple towers and high piers are prominent, few related studies have been carried out. Therefore, it is meaningful to research the stability of these bridges during their construction to reveal the impact of relevant factors on structural stability and ensure the safety and quality of the structure.

In this work, a five-tower, six-span, partial cable-stayed bridge with high piers is adopted as the engineering background, and MIDAS software is used to establish a finite element model of the entire construction stage. The structural safety and stability during the construction stage are investigated, and the sensitivity analysis of structural stability parameters is conducted. Furthermore, the impacts of the combinations of various unfavorable construction factors on the safety and stability of the structure during construction are examined. The conclusions of this research can provide information for the safety and stability analysis of partial cable-stayed bridges with multiple towers and high piers during the construction stage. This can ultimately promote the further development and application of cable-stayed bridge technology, and provide more sustainable and efficient solutions for urban transportation construction and beautification.

## 2 Structural stability evaluation theory

Stability problems are primarily respectively categorized as branch-point (first-order) and extreme-point (second-order) instability problems, the latter of which often occur in actual engineering. The solution of branch-point instability can yield the upper limit of the safety factor of extreme-point instability, thereby quickly identifying the nonlinear step size in the second-order instability problem and reducing the calculation time.

The most direct method by which to evaluate whether a structure is stable is the stability safety factor. China’s specification entitled "JTG/T 3365-01-2020 Design Specification for Highway Cable-Stayed Bridges" stipulates the stability safety factor of cable-stayed bridges [28], as follows. The structural stability safety factor of elastic buckling should not be less than 4.0, and the elastic-plastic strength stability factor, which takes into account the nonlinear effects of the material, should be no less than 2.5 for the concrete main beam and no less than 1.75 for the steel main beam.

The definition of the safety factor for structural stability mainly divides into two categories:

1. The safety factor for instability in both the construction and completed states of cable-stayed bridges is defined as the ratio between the total load capacity that the entire structure can withstand before losing stability and the design load.
$$\lambda =\frac{\left\{p_{cr}\right\}}{\left\{p_{i}\right\}}$$ (1)
  Where *λ* is the stability safety factor of the structure, {*p*_*cr*_}is the total load that can be withstood before instability under working condition *i*, i.e., the critical load of the structure, and {*p*_*i*_} is the design load under working condition *i*.
2. The total load that the entire cable-stayed bridge structure can bear before instability occurs is defined as:
$$\left\{p_{cr}\right\}=\left\{p_{d}\right\}+\lambda_{0}\left\{p_{1}\right\}$$ (2)
  Where {*p*_*d*_} represents the loads prior to condition *i*; {*p*_*i*_} represents the additional load under condition i; *λ*_0_ represents the loading of the structure at instability as a multiple of the load {*p*_*i*_}.

Currently, the stability coefficients of the structure are as follows:

$$\lambda =\frac{\left\{p_{cr}\right\}}{\left\{p_{i}\right\}}=\frac{\left\{p_{d}\right\}+\lambda_{0}\left\{p_{1}\right\}}{\left\{p_{d}\right\}+\left\{p_{1}\right\}}$$ (3)

Comparing the two calculation methods, the second method primarily considers the structure’s safety margin for additional loads, making it more suitable for comparing the safety margins of different bridge structures. The first method, on the other hand, considers the safety margin for the total load, resulting in a more conservative evaluation. This paper adopts the first method of calculating stability coefficients to evaluate the stability of the structure. The loading method for structural stability calculation is that, excluding the tension of the cables (which is loaded to the design tension), the other loads acting on the cable-stayed bridge (the self-weight of the structure and the construction load) are all incrementally loaded at the original action position at the same proportion until the structure reaches its ultimate bearing capacity. The structural stability safety factor obtained according to this loading method is relatively safe for the evaluation of the engineering structure. The calculated stability safety factor provides the structure with a certain safety reserve capacity.

## 3 Structural stability calculation conditions

### 3.1 Project overview

**Fig 1:**
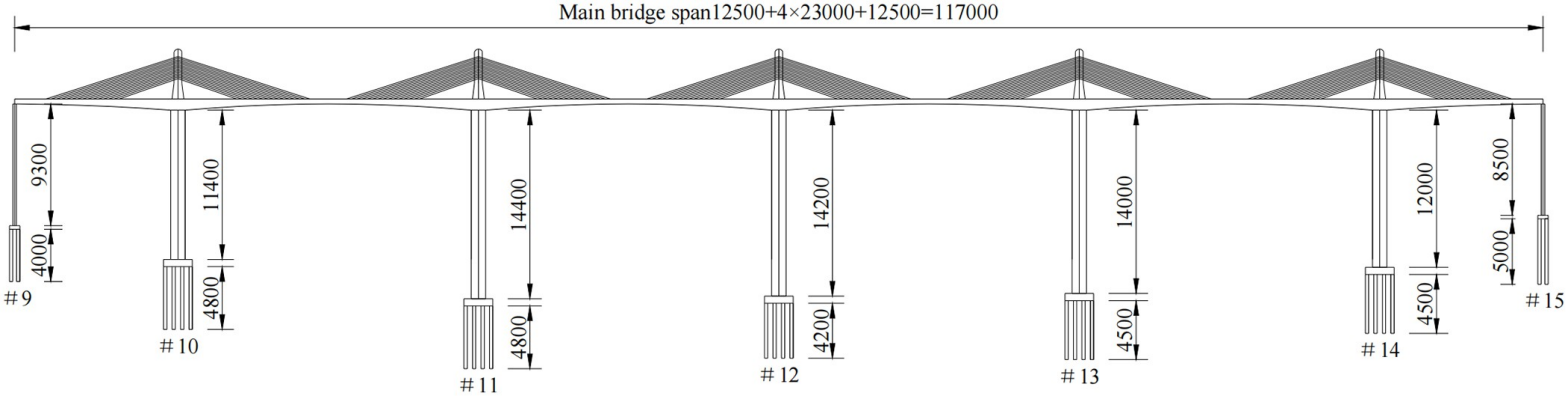
The overall layout of the main bridge(mm).

The study object of this work is a five-tower, six-span, partial cable-stayed bridge with high piers. The bridge is a prestressed concrete structure with a total length of 1170 m. The main bridge span layout is (125 + 4 × 230 + 125) m, and is a rigid frame system consolidated with tower piers and beams. The bridge deck is arranged as a two-way, four-lane deck with a total width of 29.5 m and a design speed of 100 km/h (highway level I). The overall layout of the main bridge is displayed in Fig 1.

**Fig 2:**
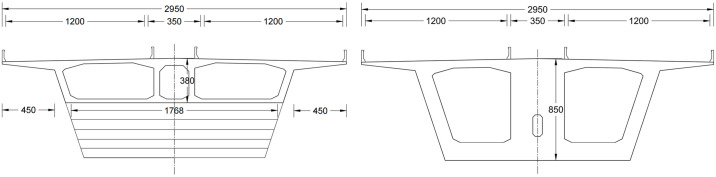
Mid-span and fulcrum cross-sections of main beam (mm).

A prestressed concrete structure is adopted for the main beam, and the form of the cross-section is a large-cantilever, variable-height, single-box, three-chamber diagonal web section. The box girder is arranged with a variable section made of C55 concrete, and the cross-sectional dimensions are presented in Fig 2.

**Fig 3:**
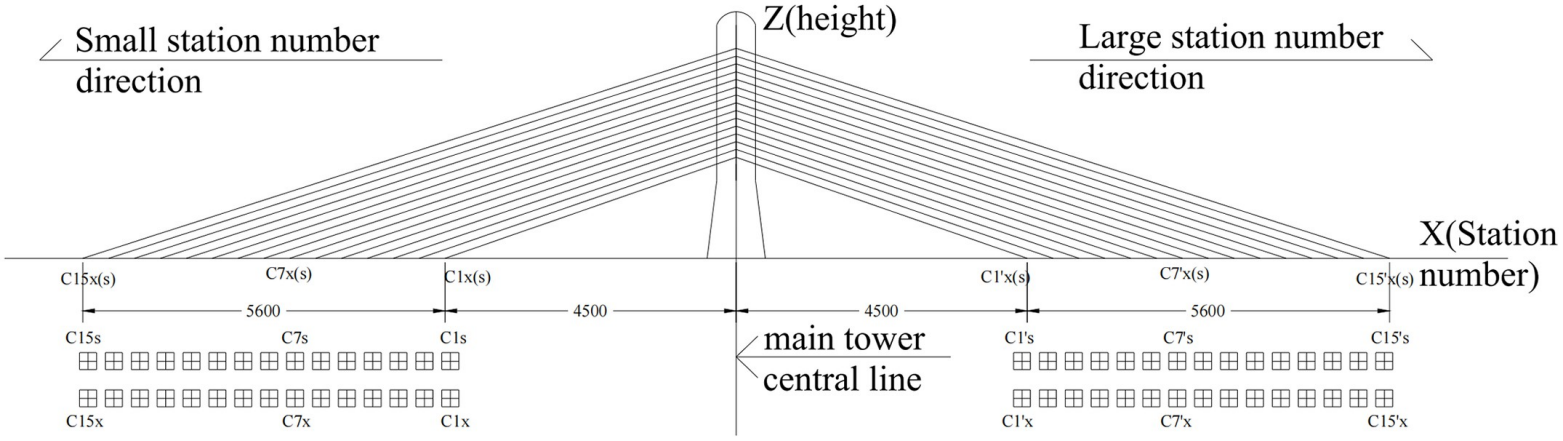
The layout of the stay cables of the main bridge.

A consolidation system is adopted for the main tower and main beam, and the height of the tower above the bridge deck is 36 m. The stay cables have a central double-cable plane and are arranged in double rows at the central separation zone of the main beam. Each cable tower is equipped with 15 pairs of stay cables (a total of 150 along the whole bridge). The layout of the stay cables is presented in Fig 3. The main pier is a double thin-walled hollow pier in an octagonal shape. The distance between the two limbs is 2 m, and the main pier heights are 114 m (#10), 144 m (#11), 142 m (#12), 140 m (#13), and 120 m (#14).

### 3.2 Finite element model

**Fig 4:**
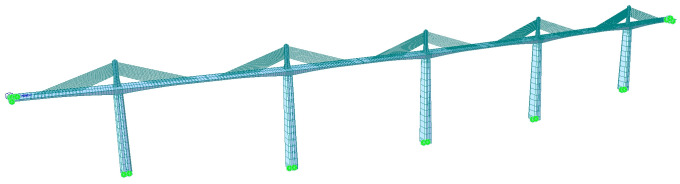
The overall model of the full bridge.

The large-scale bridge structural analysis software MIDAS/CIVIL [3] was used to simulate and model a five-tower, six-span, partial cable-stayed bridge in Shaanxi Province, China. The overall model of the whole bridge exhibited in Fig 4 has a total of 1,311 nodes and 1,138 units, among which the main beam, main pier, and main tower are simulated by beam elements, and the stay cables are simulated by cable elements. The actual longitudinal slope is considered for the main beam, for which a single main beam model is adopted. The stiffness and mass of the bridge deck system are evenly distributed to the beam elements corresponding to the main beam. The main beam and the cable stay are rigidly connected in a master-slave relationship. The main beam, main pier, and main tower are rigidly connected to simulate the consolidation of the tower, pier, and beam. General support is adopted for the bottom of the main pier to restrain translation and rotation in all directions, i.e., fixed restraint.

### 3.3 Construction stages and calculation conditions

**Fig 5:**
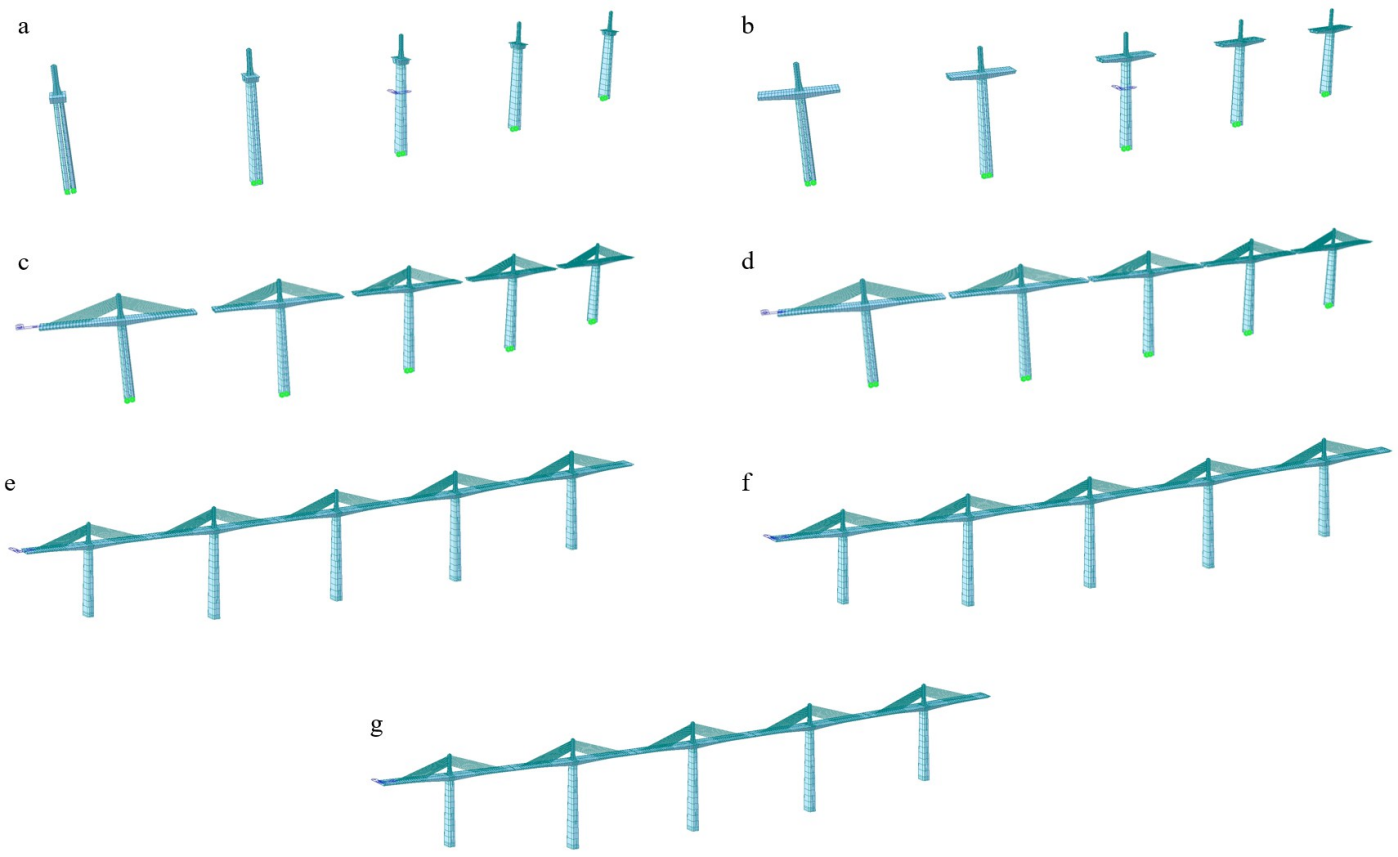
The schematic diagrams of the main construction stages. (a) Bare tower construction. (b) Maximum cantilever construction without cables. (c) Maximum cantilever construction with cables. (d) Side-span closure. (e) Secondary mid-span closure. (f) Mid-span closure. (g) Second-phase paving.

The construction process of the main girder structure of this bridge is divided into 87 construction stages, and linear elastic and nonlinear stability analyses were carried out for seven main construction stages. These include bare tower construction (CS1), maximum cantilever construction without cables (CS22), maximum cantilever construction with cables (CS71), side-span closure (CS76), secondary mid-span closure (CS80), mid-span closure (CS84), and second-phase paving (CS87). The main construction stages are shown in Fig 5 and are detailed as follows:

① *CS1*, *bare tower construction*: the main tower and cable saddle are constructed;
② *CS22*, *maximum cantilever construction without cables*: the main beam is symmetrically cantilevered and poured to the beam section before the cable;
③ *CS71*, *maximum cantilever construction with cables*: the stay cables are erected and tensioned, the hanging basket is moved forward, and this process is repeated until the final beam section is reached;
④*CS76*, *side-span closure*: the hanging basket is dismantled, the side-span closure hanging basket is installed, and the pouring of the side-span closure section is completed;
⑤ *CS80*, *secondary mid-span closure*: the secondary mid-span closure hanging basket is installed at the same time as the secondary mid-span side counterweights, and the pouring of the secondary mid-span closure section is completed;
⑥*CS84*, *mid-span closure*: the mid-span closure basket is installed at the same time as the mid-span side counterweight, and the pouring of the mid-span closure section is completed;
⑦ *CS87*, *second-phase paving*: the bridge deck system is constructed.

The loads borne by the bridge during the construction stage are mainly dead load, stay cable force, prestressed load, and construction-stage load, the values of which are reported in Table 1.

**Table 1:** The calculated loads.

Load	Load size	
Prestressed load	1395 MPa	
Stay cable force	4700 kN	
Construction load	Hanging basket load (including formwork and machinery)	2000 kN
	Gondola load (including formwork and machinery)	1000 kN
	Side-span closure weight	262.6 kN
	Secondary mid-span closure counterweight	175.1 kN
	Mid-span closure weight	175.1 kN

### 3.4 Material specifications

The main pier is made of C50 concrete, and the main tower and main beam are made of C55 concrete. Considering the influence of reinforcement, the concrete bulk weight is calculated according to 26kN/m^3^. The second stage pavement load is 164kN/m, and the unit uniform load is added to the main beam. The cable-stayed cable system adopts steel strand cable-stayed cable. According to the design draw ing, the cable-stayed cable force is 4700KN. The material parameters are shown in Table 2.

**Table 2:** Material specifications.

Material material	elasticity modulus(MPa)	poisson ratio	unit weight(KN/m^3^)
C50	3.45×10^4^	0.2	26
C55	3.55×10^4^	0.2	26
steel strand	1.95×10^5^	0.3	78.5

### 3.5 Finite element model verification

**Fig 6:**
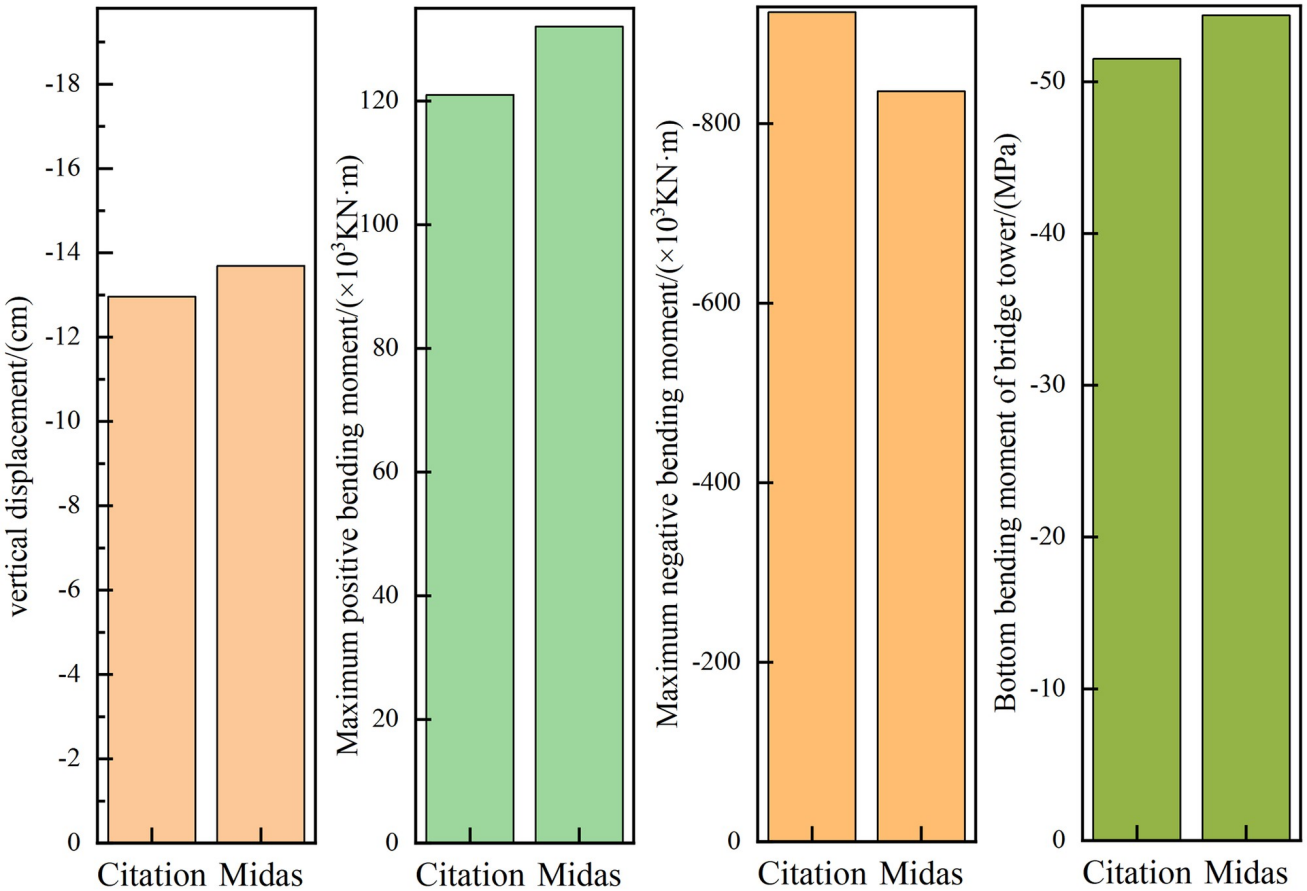
Key calculation data comparison.

**Fig 7:**
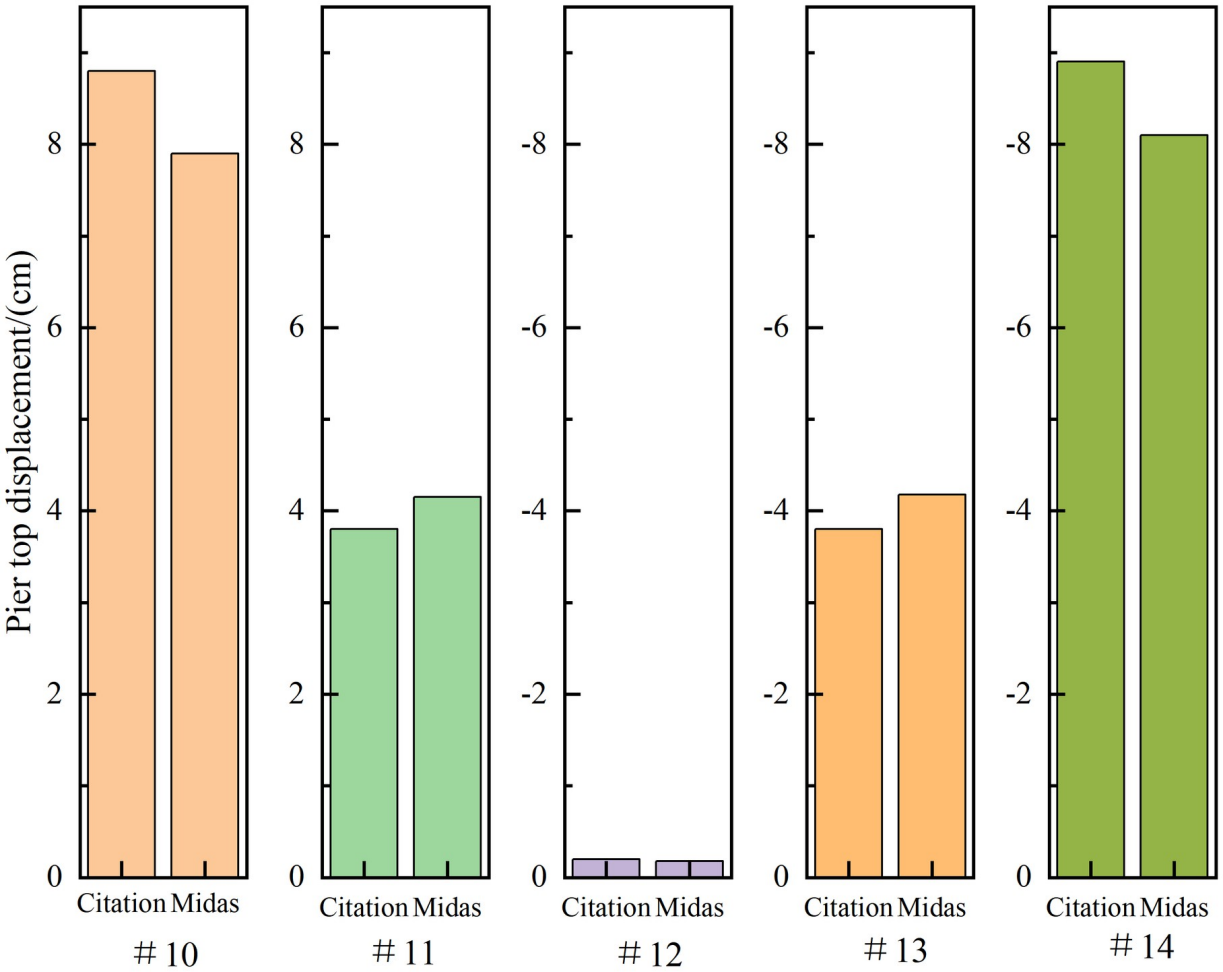
Comparison of pier top displacement.

In order to verify the accuracy of the finite element model, the maximum vertical deflection, maximum positive and negative bending moment, tower root moment and pier top displacement of the bridge are compared by referring to the calculation data of the same bridge in the existing literature [29, 30]. Among them, the comparison results of maximum vertical deflection, maximum positive and negative bending moment, and bending moment at the tower root are shown in Fig 6, and the comparison results of displacement at the top of each pier are shown in Fig 7.

By comparing the Midas calculation results with the key calculation data in the citation, the difference between the maximum vertical deflection of the bridge and the maximum positive and negative bending moments of the bridge is 5.63% and 9.15% and -9.48% respectively. The bending moment difference at the bottom of the bridge tower is 6.28%. The difference of displacement of each pier is -9.97%, 9.21%, -10%, 10% and -8.98%, respectively. By comparison, it is found that the difference between Midas calculation results and the key data in the citation is within ±10%. It can be seen that the established finite element model has a certain accuracy, which provides a basis for the accurate calculation of structural stability in the following paper.

### 3.6 Research concept

A finite element model of the entire construction process of a five-tower, six-span, partial cable-stayed bridge was constructed based on the real project, and the key construction stages were subjected to linear elastic and geometric nonlinear stability analyses. Based on the stability analyses, the stage during which the stability problems are the most prominent was identified, after which the impacts of various influencing factors on the stability of the structure during construction were analyzed. The impacts of changes in the structural parameters (the main tower stiffness, main pier stiffness, and main beam stiffness) on the safety and stability of the structure during construction were analyzed, as were the impacts of the multi-factor combination of the reduced stiffness of the main pier and two unfavorable construction factors (the detachment of the construction hanging basket and the asymmetric construction of cantilever pouring on both sides of the main beam).

## 4 Stability studies

**Fig 8:**
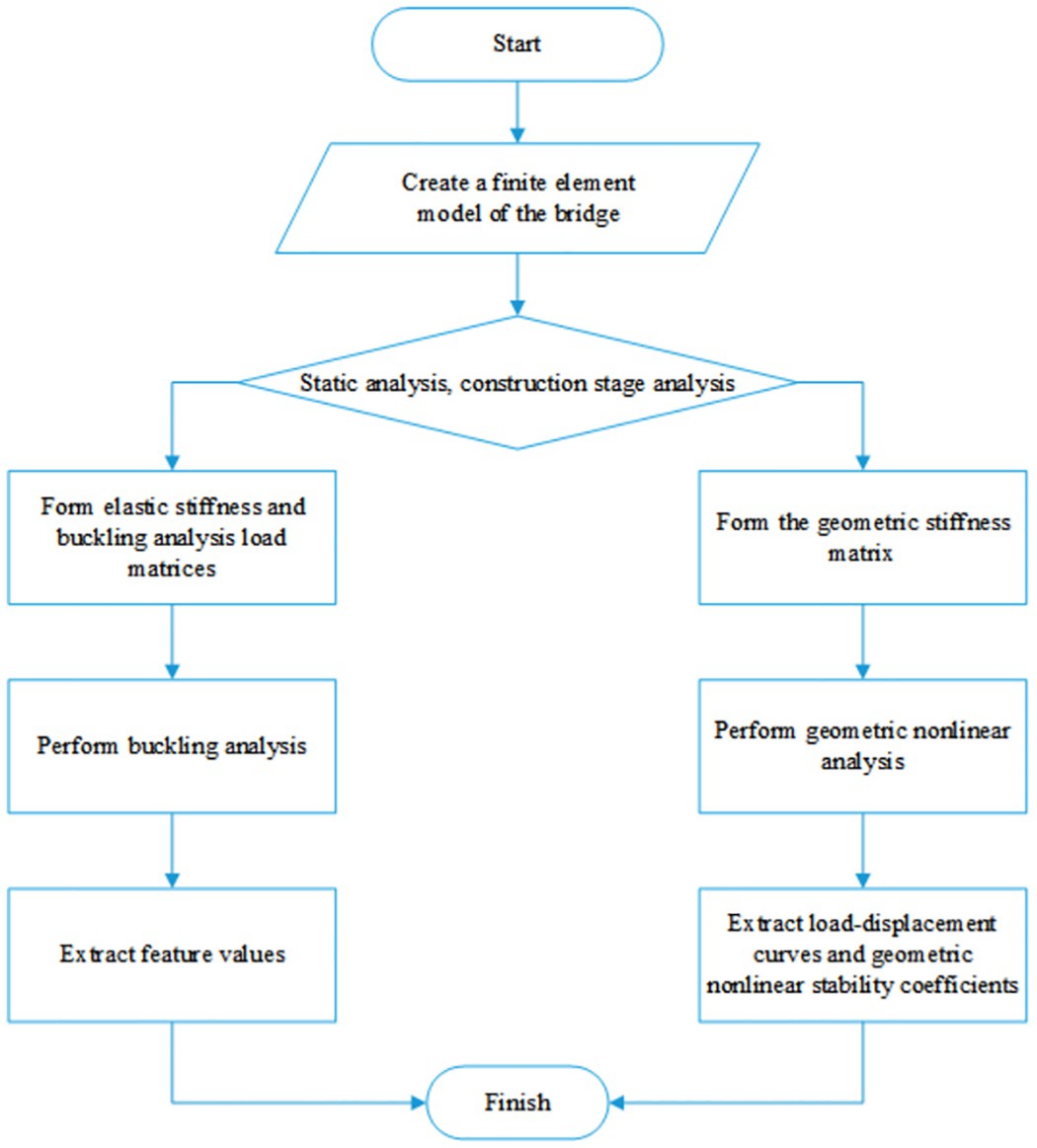
The technical route of the structural stability analysis.

Linear elastic stability analysis, also known as eigenvalue buckling analysis, does not consider the influence of structural nonlinearity. However, during the construction of long-span partial cable-stayed bridges with high piers, the influence of structural geometric nonlinearity is prominent. Therefore, when conducting the stability analysis of such bridges during the construction process, both linear elasticity and geometric nonlinearity should be considered. For partial cable-stayed bridge structures, plastic deformation is generally not permitted during construction, and the structural systems all work within the linear range. Therefore, in this work, the nonlinear problems of partial cable-stayed bridges are primarily studied based on the geometric nonlinearity of the structure [31]. The influence of geometric nonlinearity mainly manifests as large displacement, sag, and beam-column effects. The bridge structure investigated in this article was analyzed at various key construction stages in consideration of these three effects, including the cumulative effect of displacement and stress. Geometric nonlinear analysis was performed on the structure, and the technical route of the structural stability analysis is shown in Fig 8.

### 4.1 Construction stage stability

Finite element software was used to conduct the linear elastic and nonlinear stability analyses of the structure and determine the corresponding stability safety factors and instability modes at each stage.

**Fig 9:**
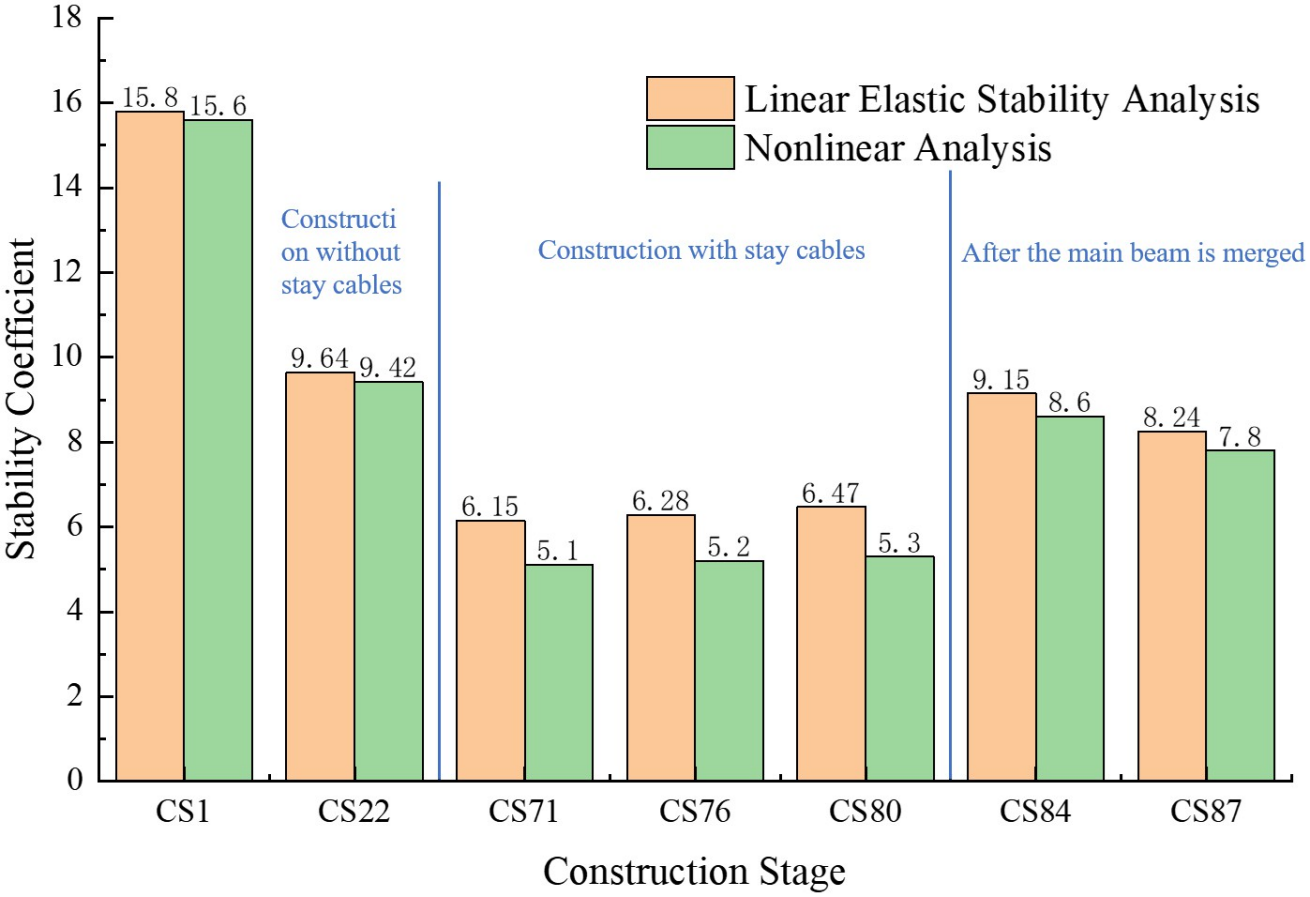
The structural stability coefficients during each construction stage.

**Fig 10:**
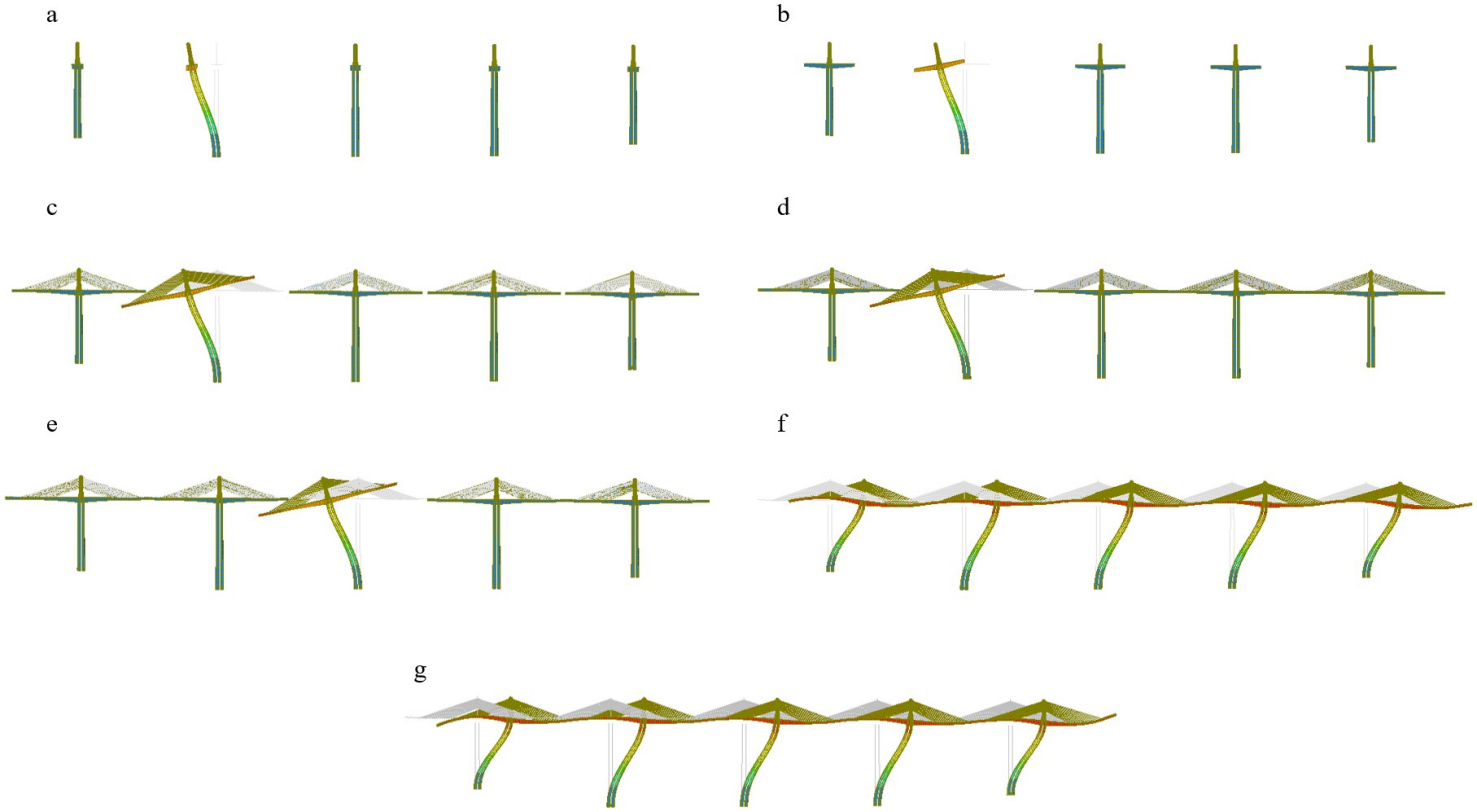
The first-order instability modes in each construction stage. (a) CS1, Bare tower construction. (b) CS22, Maximum cantilever construction without cables. (c) CS71, Maximum cantilever construction with cables. (d) CS76, Side-span closure. (e) CS80, Secondary mid-span closure. (f) CS84, Mid-span closure. (g) CS87, Second-phase paving.

Among the various modes of structural instability, the first-order instability mode occurs first, is the most likely to occur, and is the most important. The stability coefficient corresponding to the first-order instability mode at each construction stage is the value for that stage; thus, it determines the safety and stability of the bridge at each stage. Therefore, the first-order stability coefficients and instability modes of each construction stage are of primary analytical concern, and are respectively presented in Figs 9 and 10.

The following conclusions can be drawn from Figs 9 and 10.

(1) From stage CS1 to stage CS22, the geometric nonlinear stability coefficient of the structure was found to be respectively reduced by 1.27% and 2.28% as compared with the linear elastic stability coefficient of the structure at the same stage; thus, the values are almost the same. The reason for this is that during the symmetrical cantilever pouring of the main beam, the main pier and the main tower of the structure are consolidated, which improves the stiffness of the structure. During the cable construction stage, due to the continuous increase of the cantilever end, the structural geometric nonlinearity is prominent, and the nonlinear stability coefficient at this stage was found to be at least 17.07% lower than the linear elastic stability coefficient. After the main beam is closed, the structural system changes, and the structural integrity and stiffness increase.

In comparison with their linear elastic stability coefficients, the geometrically nonlinear stability coefficients for the two construction stages, CS84 and CS87, are reduced by 6.01% and 5.34%, respectively, thus reflecting lower decreases. Therefore, when the main beam is constructed with fewer beam sections, the impact of the geometric nonlinearity of the structure is minimal. Furthermore, after the main beam is closed, the impact of the geometric nonlinearity of the structure is small. In contrast, in the construction section in which the main beam is not closed, the influence of geometric nonlinearity is significant.

(2) From stage CS1 to stage CS71, the linear elastic and geometric nonlinear stability coefficients of the structure were found to decline. After the main beam begins to close, the structural system changes, and the overall stability of the bridge structure is improved; moreover, the linear elastic and geometric nonlinear stability coefficients were also found to increase. In stage CS71, the linear elastic and nonlinear stability coefficients were respectively found to be 6.15 and 5.1, which are smaller than those in the other construction stages. Therefore, stage CS71 was identified as the most unfavorable construction stage of the structure and requires targeted attention during construction. Appropriate measures must be taken to ensure its construction safety. The impacts of relevant factors on the stability of the structure in this stage are explored later in the article.

(3) The bridge structure begins with the cantilever pouring of the main beam from stage CS1 to stage CS76, and, in this process, pier #11 is the most unfavorable. At each stage, pier #11 is the first to experience longitudinal instability. During stage CS80, the secondary mid-span closure increases the stiffness of the structure, so the first-order instability is the longitudinal instability of pier #12. From stage CS84 to stage CS87, the structure merges into a whole structure, and the first-order instability mode is the overall longitudinal instability failure of piers #10 to #14.

(4) During the entire construction of the bridge structure, the linear elastic stability coefficient at each stage was found to be greater than 4.0, and the nonlinear stability coefficient was greater than 2.5; these values meet the stability analysis requirements of the relevant code.

### 4.2 Seismic stability

**Fig 11:**
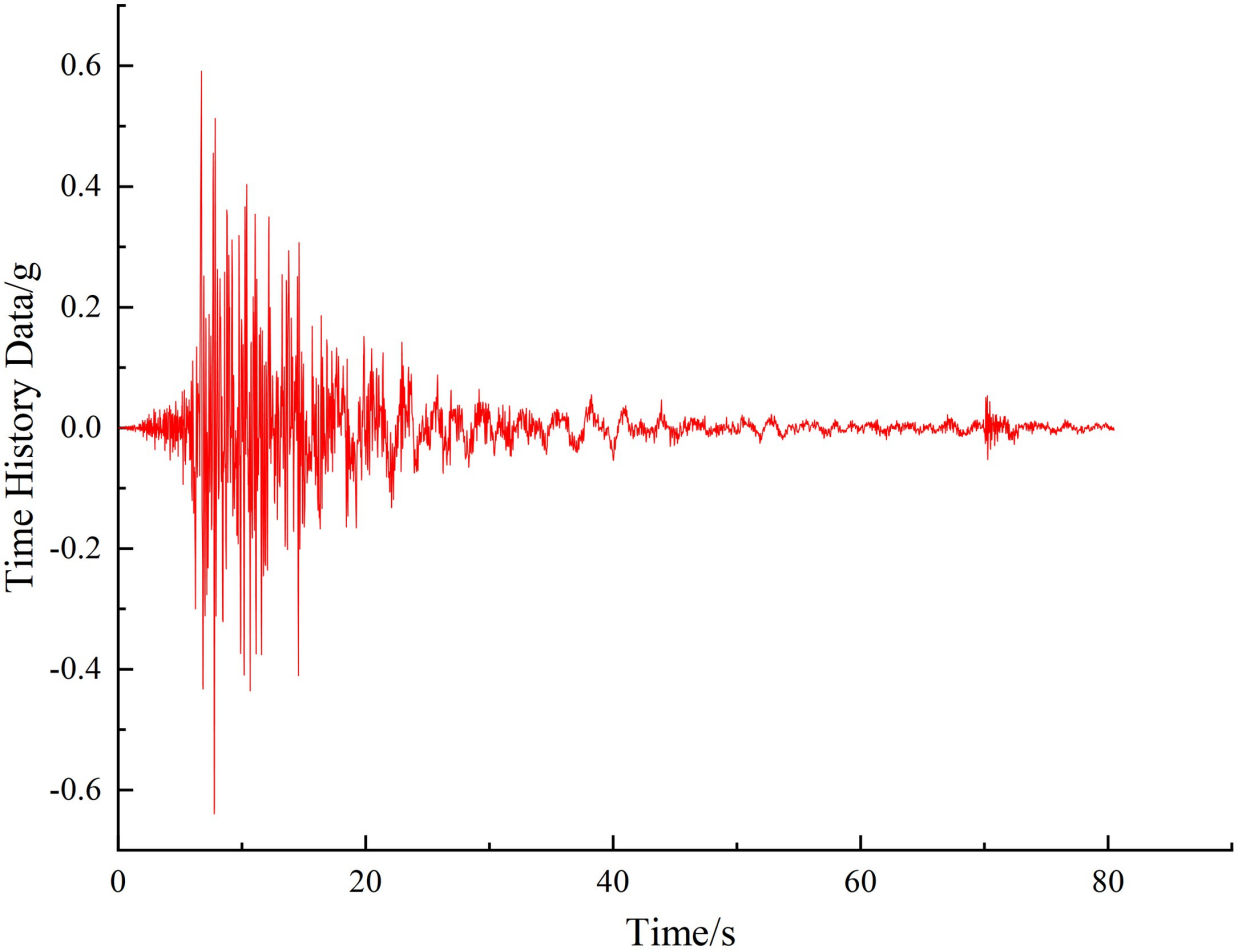
Seismic load.

Regarding the seismic loads that may exist during the construction of bridges, analyze the construction stage stability of the structure under these seismic loads. Due to the low probability of earthquakes and the moderate seismic intensity at the bridge site, the seismic analysis of the structure is conducted using the Bridge E1 earthquake, with a peak ground acceleration of 0.105g and a characteristic period of 0.5 seconds. The seismic amplification factor curve is shown in Fig 11.

**Fig 12:**
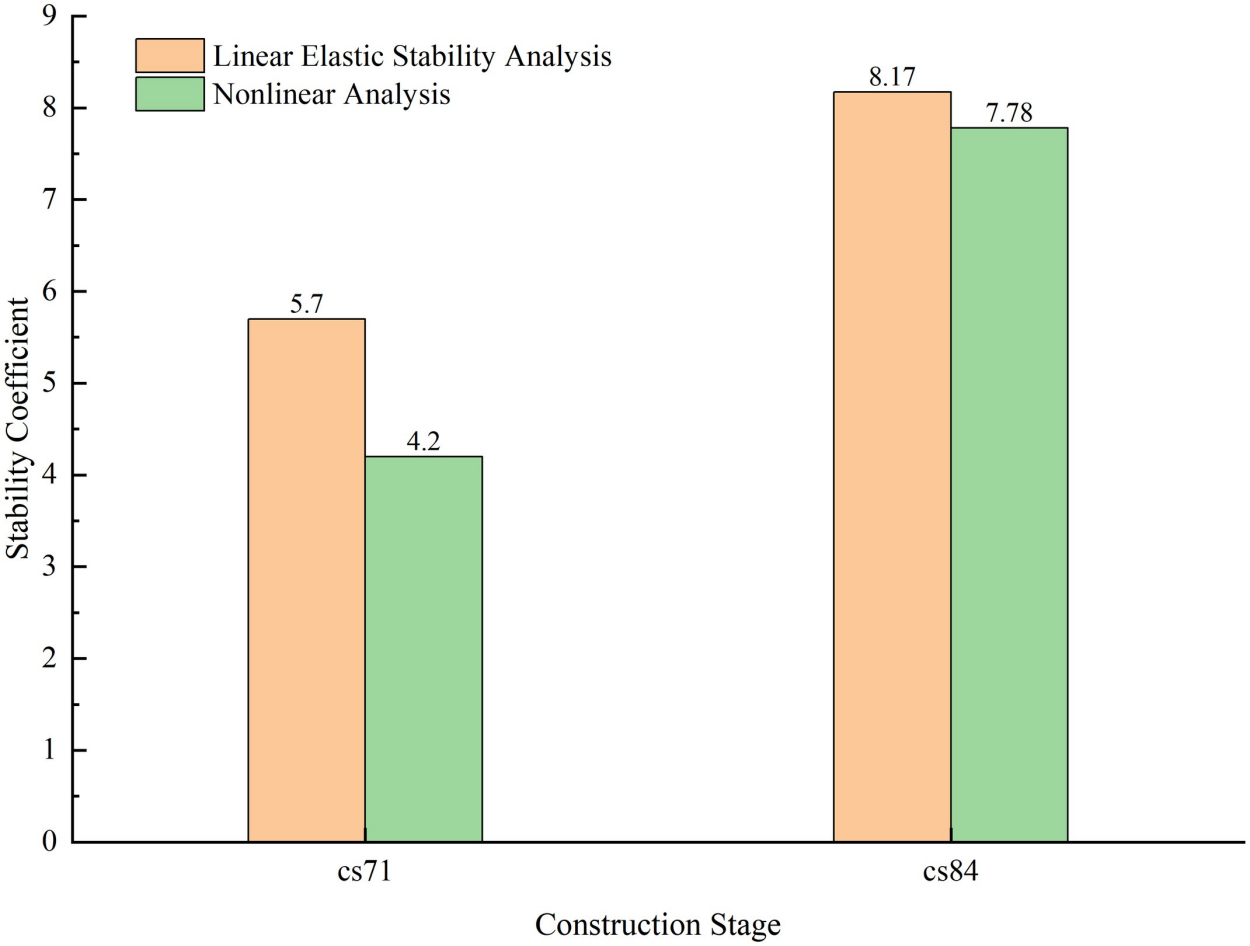
Stability under seismic loads.

When analyzing the stability of construction stages under seismic loads, keeping the seismic load constant, an assessment of the stability of loads during construction stages is conducted. From stability analysis, it is evident that the stage with the longest cantilever with cables is the most unfavorable construction stage for the structure, and after the mid-span closure, the structural system will change. Therefore, stage CS71 with the longest cantilever with cables and CS84 after the mid-span closure were chosen for stability analysis under seismic loads. Each stage’s stability coefficient is shown in Fig 12.

Based on Fig 12, it can be observed that under seismic loads, the linear elastic and nonlinear stability coefficients of stage CS71 with the longest cantilever with cables are 5.82 and 4.2 respectively. Compared to the same stage, these coefficients have decreased by 7.3% and 17.65% respectively. For stage CS84 after the mid-span closure, the coefficients are 8.17 and 7.78, showing reductions of 10.71% and 9.54% respectively.

It is evident that seismic loads reduce the stability coefficients during construction stages. However, all stages maintain a linear elastic stability coefficient greater than 4.0 and a nonlinear stability coefficient greater than 2.5, which satisfies regulatory requirements. Therefore, seismic loads do influence the stability of construction stages, with the stage involving the longest cantilever with cables remaining the most critical construction phase.

## 5 Study on the influence of the design parameters

Numerous factors affect the stability of partial cable-stayed bridges with multiple towers and high piers during their construction. To explore these factors, the impacts of the stiffnesses of the main tower, the main beam, and the main pier on the structural stability are first determined. By appropriately reducing and increasing these values, their impacts on stability are judged via the stability coefficients at each stage. Then, the impacts of the combinations of the reduced stiffness of the main pier with two unfavorable construction factors on the stability of the construction stage are further determined.

### 5.1 Design parameter sensitivity analysis

#### 5.1.1 Influence of changes in the stiffness of the main tower

**Fig 13:**
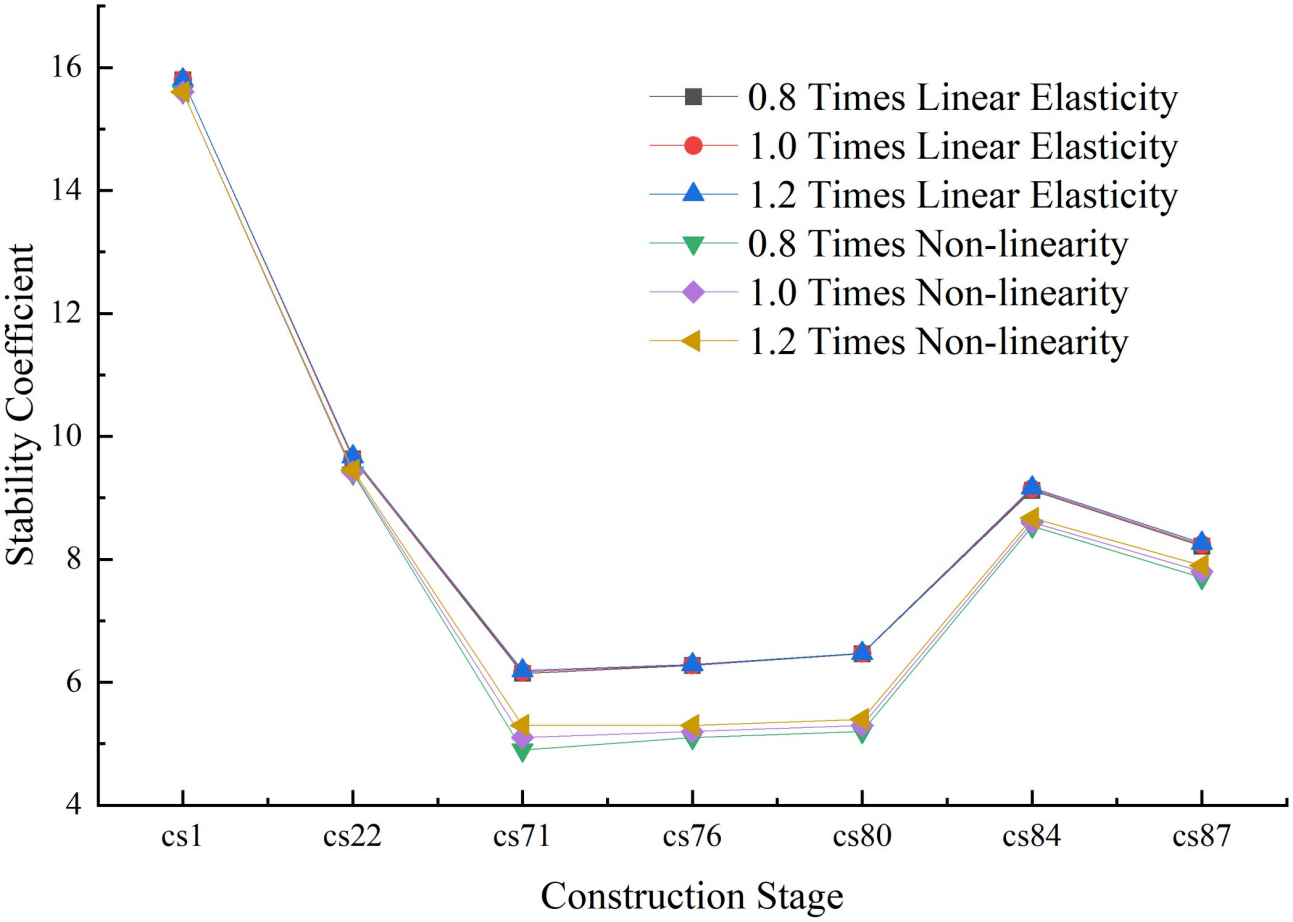
The influence of the stiffness of the main tower.

The stiffness of the main tower will be deviated due to construction errors and changes in live load conditions. In order to comprehensively analyze the influence of stiffness fluctuations that may be encountered in actual engineering scenes, this paper studies the influence of the main tower stiffness on structural stability by changing the elastic modulus to 0.8 times, 1.0 times and 1.2 times of the original value. By choosing these three specific multiples, a reasonable range of stiffness variations can be included, ensuring that no critical conditions that can affect the performance of the structure are overlooked. The foundation stiffness of the main tower is 3.55×10^7^ *kN*/*m*^2^. Fig 13 presents the resulting structural safety and stability coefficients of partial cable-stayed bridges during construction.

The stiffness of the main tower was found to have little effect on the linear elastic stability coefficient during the construction stage, and the variation range was within 2%. The geometric nonlinearity of the structure caused by changes in the stiffness of the main tower was more obvious during stage CS71. As the stiffness of the main tower increased, the nonlinear stability coefficients increased by 4.10% and 3.92%, respectively. However, in the other construction stages, changes in the stiffness of the main tower were found to have indistinctive impacts on the structure, and the range of change was within 2%. With the change of the stiffness of the main tower, the linear elastic and nonlinear stability coefficients of the bridge structure underwent limited changes. Thus, this factor was determined to have little impact on the stability of the structure during construction.

#### 5.1.2 Influence of changes in the stiffness of the main pier

**Fig 14:**
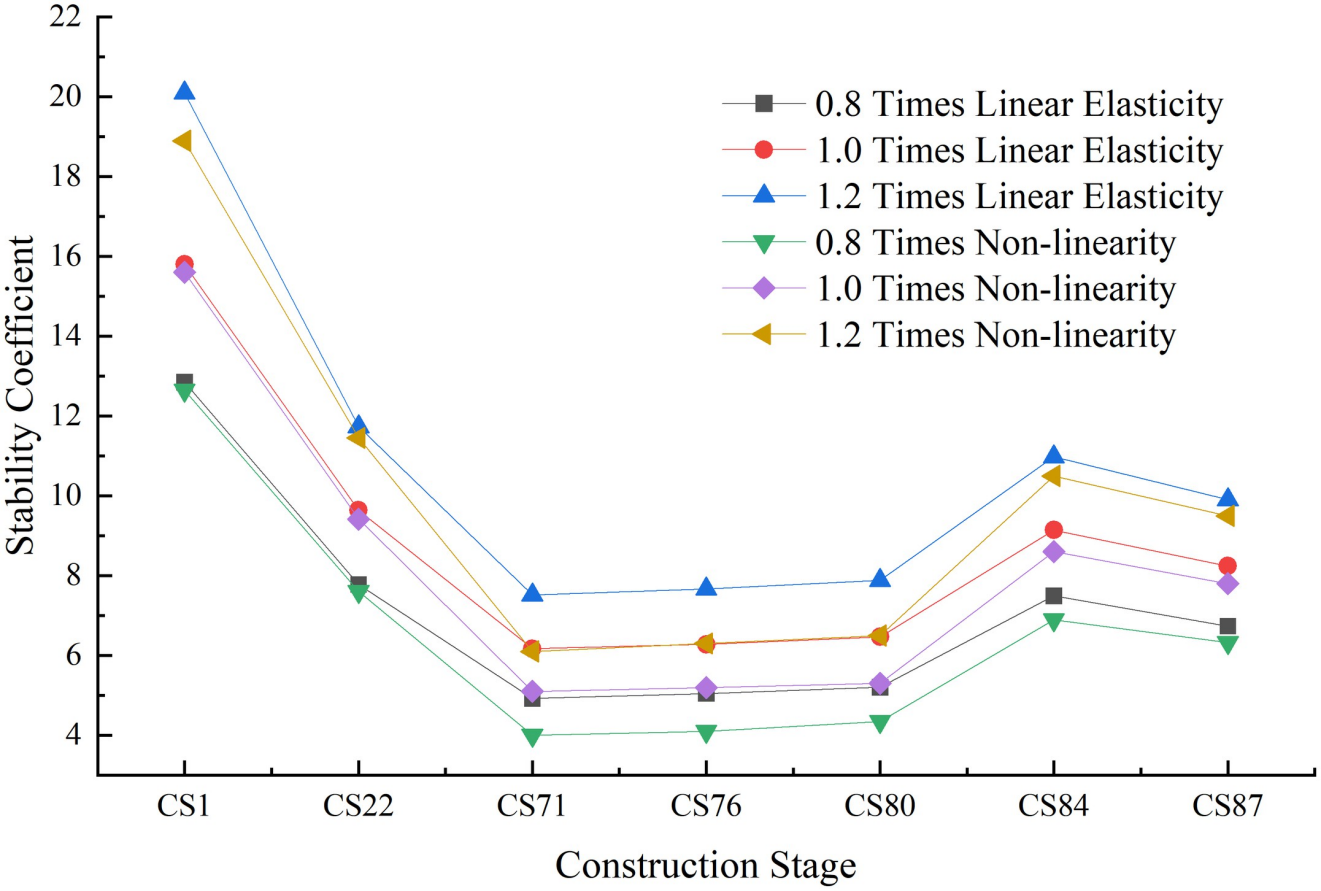
The effects of the stiffness of the main pier.

For cable-stayed bridges with high piers, it is necessary to study the stiffness of the main pier to determine the stability of the structure during construction. The stiffness value was respectively set as 0.8, 1.0, and 1.2 times the stiffness of the main pier. The foundation stiffness of the main pier is 3.45 × 10^7^ *kN*/*m*^2^. Fig 14 presents the structural safety and stability coefficients of the partial cable-stayed bridges during the construction stage.

The stiffness of the main pier was found to have a significant impact on the linear elastic stability coefficient during the construction stage; at each stage, the coefficient was proportional to the main pier stiffness. With the change in the stiffness value from 0.8 times the stiffness of the main pier to 1.0 to 1.2 times the value, the linear elastic stability coefficient of the structure respectively increased by a maximum of 25.25% and 21.82%. Changes in the stiffness of the main pier also had significant impacts on the geometric nonlinear stability coefficient of the structure; as the stiffness increased, the coefficient increased in each stage. The most obvious change occurred during stage CS71; as the main pier stiffness increased, the nonlinear stability coefficient respectively increased by 27.5% and 20.10%. Thus, due to the substantial changes in the linear elastic and nonlinear stability coefficients, the stiffness of the main pier is an important factor affecting the stability of the structure during construction.

#### 5.1.3 Influence of changes in the stiffness of the main beam

**Fig 15:**
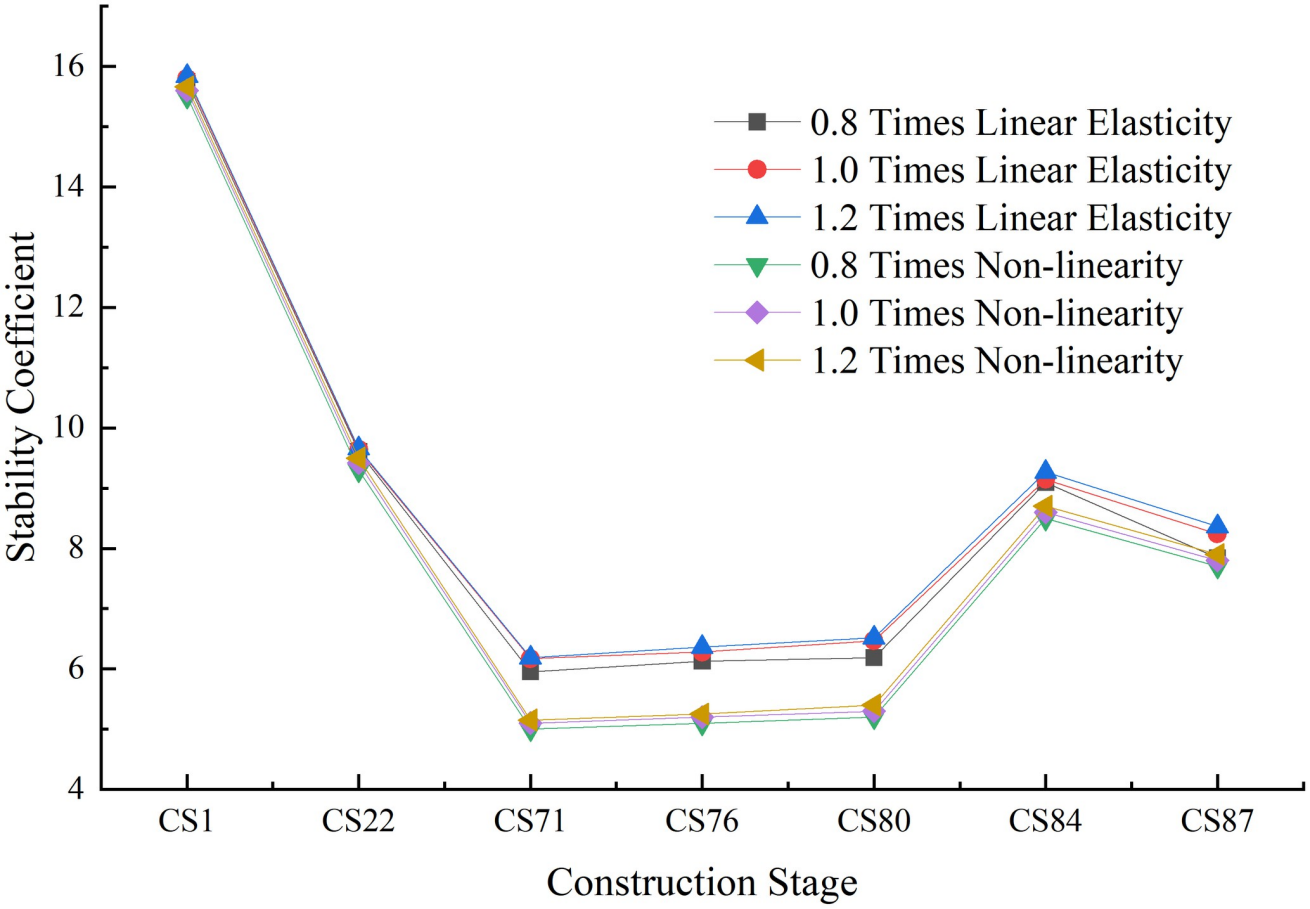
The effects of the stiffness of the main beam.

The stiffness of the main beam is also an important design parameter for partial cable-stayed bridges. The stiffness value was respectively set as 0.8, 1.0, and 1.2 times the stiffness of the main beam. The foundation stiffness of the main pier is 3.55 × 10^7^ *kN*/*m*^2^. Fig 15. presents the resulting structural safety and stability coefficients of the partial cable-stayed bridges during construction.

The stiffness of the main beam was found to have little impact on the linear elastic stability coefficient during construction. As the stiffness of the main beam increased, the linear elastic stability coefficient of the structure increased slightly to maximum values of 5.04% and 1.54%, respectively. Changes in the stiffness of the main beam also had little impact on the geometric nonlinearity of the structure; as the stiffness increased, the growth rate of the nonlinear stability coefficient remained within 2%. This is because the instability mode of the structure is the longitudinal instability failure of the main pier, on which the stiffness of the main beam has little impact. Therefore, overall, the stiffness of the main beam was determined to have little impact on the stability of the structure during the construction process.

Based on the stability analysis, the stiffnesses of the main beam and main tower were found to have little impact on the stability of the structure during construction, while the stiffness of the main pier was found to have a significant impact. The results indicate that during the construction of partial cable-stayed bridges, the main pier has the greatest impact on the safety and stability of the structure. The construction and management of the main pier should thus be strengthened to ensure the strength and stiffness of the pier structure, which can improve the safety reserve value of the bridge structure during construction.

### 5.2 Cross-influence of multiple factors

**Fig 16:**
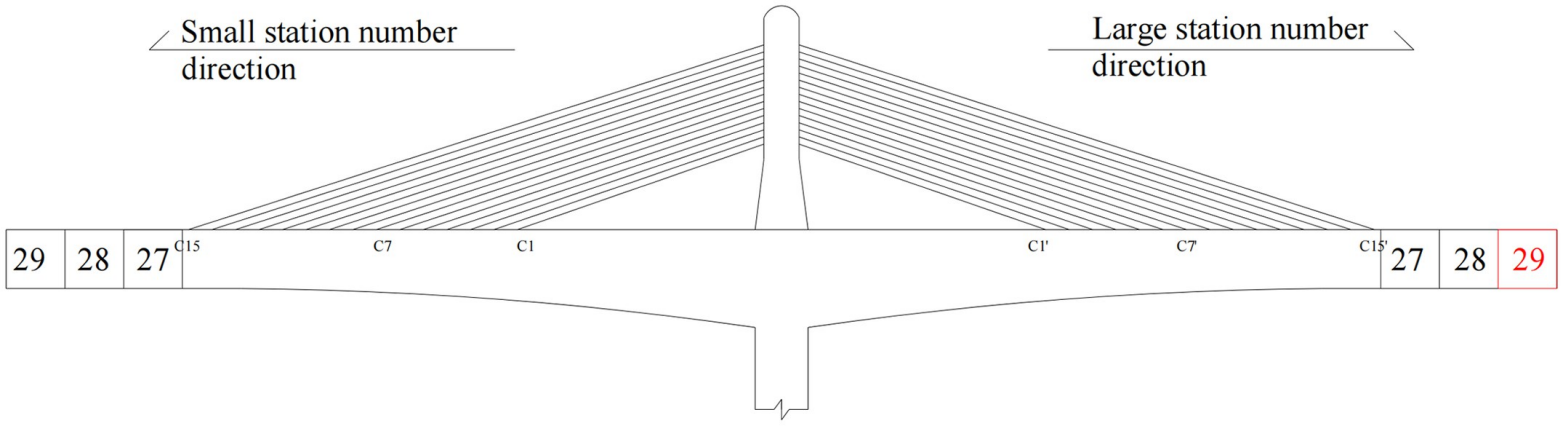
The schematic diagram of asymmetric construction in stage CS71.

Based on the sensitivity analysis, the safety and stability of the structure during construction were determined to be highly sensitive to the main pier stiffness. Furthermore, the asymmetric construction of cantilever pouring on both sides of the main beam and the detachment of the construction hanging basket are important influencing factors of stability during construction. Therefore, a stability analysis was conducted based on combinations of these unfavorable factors, i.e., the reduction in the stiffness of the main pier, the asymmetric construction of cantilever pouring on both sides of the main beam, and the detachment of the construction hanging basket. Fig 16 presents the schematic diagram of the asymmetric construction of the main beam in the stage of maximum cantilever construction with cables.

The preceding investigations revealed that the stage of maximum cantilever construction with cables (CS71) has the lowest stability coefficient, i.e., it is the most unfavorable construction stage. Therefore, the stability analysis of the multi-factor combination for stage CS71 was carried out, and the analysis conditions are reported in Table 3.

**Table 3:** The working conditions for the stability analysis.

Combination	Working conditions
1	Asymmetric construction of the main beam and the reduction of the stiffness of the main pier
2	Detachment of the hanging basket and the reduction of the stiffness of the main pier

#### 5.2.1 The stiffness of the main pier is reduced and the main beam is constructed asymmetrically

**Fig 17:**
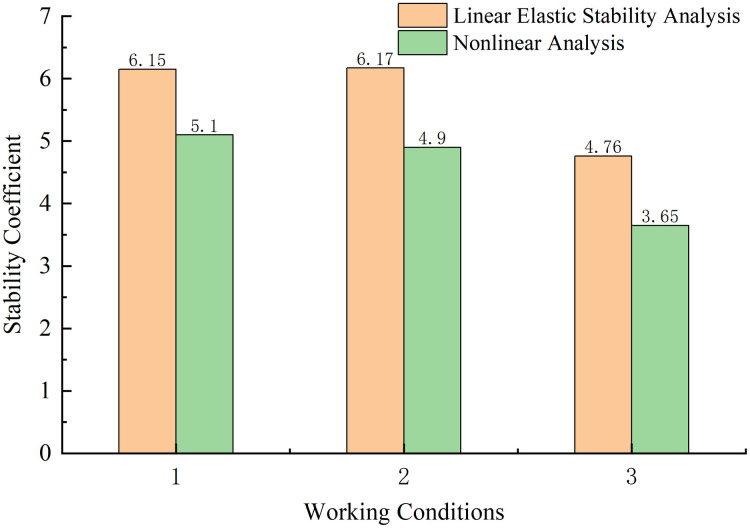
Stability coefficient under different working conditions.

The piers of the structure considered in this study are relatively high, and the pier stiffness has a significant impact on the structural stability during construction. Therefore, a stability analysis was carried out under the reduction in the stiffness of the main pier and asymmetric cantilever construction on both sides of the main beam. The impact of asymmetric construction on the structural stability was analyzed under values of 0.8 and 1.0 times the stiffness of the main pier. The analysis conditions are shown in Table 4, and the analysis results are demonstrated in Fig 17.

**Table 4:** The analysis conditions.

Working condition	Construction stage	Working conditions
1	Maximum cantilever construction with cables (CS71)	The stiffness was 1.0 times the stiffness of the main pier; Symmetrical cantilever construction was carried out on both sides of the main beam to block 29.
2		The stiffness was 1.0 times the stiffness of the main pier; Asymmetrical construction was carried out on both sides of the main beam from the beginning: the cantilever pouring of the main beam on the left side was carried out to block 29, while that on the right side was carried out to block 28.
3		The stiffness was 0.8 times the stiffness of the main pier; Asymmetrical construction was carried out on both sides of the main beam from the beginning: the cantilever pouring of the main beam on the left side was carried out to block 29, while that on the right side was carried out to block 28.

According to Fig 17, based on the comparison of working conditions 1 and 2, the asymmetric construction of the main beam was found to increase the linear elastic stability coefficient of the structure by 0.33%, reflecting a small impact, while the nonlinear stability coefficient was decreased by 3.92%.

Based on the comparison of working conditions 2 and 3, when the main beam was constructed asymmetrically, the reduction of the main pier stiffness was found to greatly affect the stability of the structure. When the stiffness of the main pier was reduced to 0.8 times the value, the linear elasticity and nonlinear stability coefficients of the structure were reduced by 22.85% and 25.51%, respectively, which are very large decreases. This fully demonstrates that the decrease of the stiffness of the main pier when the main beam is constructed asymmetrically will greatly reduce the construction stability of the superstructure of the bridge.

Based on the comparison of working conditions 1 and 3, the asymmetric construction of the main beam under the reduction of the stiffness of the main pier to 0.8 times the value was found to significantly reduce the stability of the structure in construction stage CS71; the linear elasticity and nonlinear stability coefficients respectively decreased by 22.61% and 28.43%.

The analysis shows that the instability mode under the three working conditions in stage CS72 was the longitudinal instability of pier #11, and the first-order instability mode is shown in Fig 10c. The combined effect of the reduced stiffness of the main pier and the asymmetric construction of the main beam was found to have little impact on the structural instability mode. Therefore, in the stage of maximum cantilever construction with cables, these working conditions will individually greatly reduce the construction stability of the structure, and their combined effect will further decrease the stability coefficient, thereby increasing the risk of structural instability. These conditions are detrimental to construction and should be avoided.

#### 5.2.2 The stiffness of the main pier is reduced and the construction hanging basket of the main beam detaches

**Fig 18:**
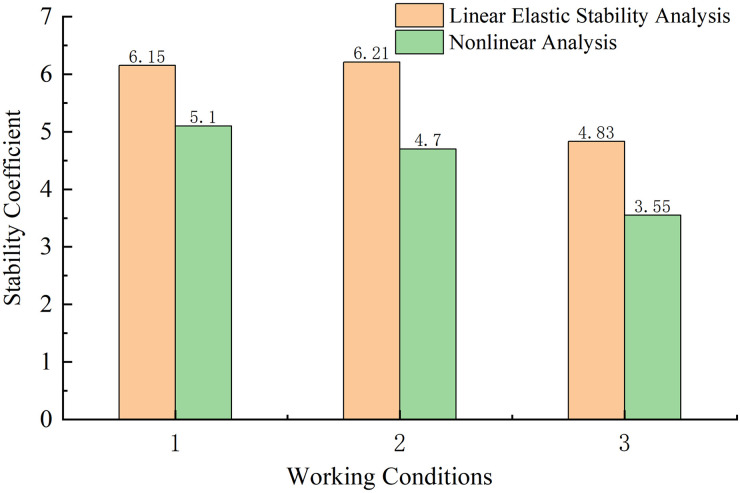
Stability coefficient under different working conditions.

A stability analysis was conducted under the conditions of the hanging basket detaching during the cantilever construction of the main girder and the reduction of the stiffness of the main pier. Values of 0.8 and 1.0 times the stiffness of the main pier were adopted to analyze the detachment of the hanging basket during the cantilever construction of the main beam. The working conditions are reported in Table 5, and the analysis results are exhibited in Fig 18.

**Table 5:** Calculation conditions when the stiffness of the main pier decreases and the construction hanging basket of the main beam detaches.

Working condition	Construction stage	Working conditions
1	Maximum cantilever construction with cables (CS71)	The stiffness was 1.0 times the stiffness of the main pier; The main beam cantilevered with cables was poured until block 29, and the hanging basket did not fall off.
2		The stiffness was 1.0 times the stiffness of the main pier; The main beam cantilevered with cables was poured until block 29, at which time the right hanging basket and the main beam block 12 simultaneously detached.
3		The stiffness was 0.8 times the stiffness of the main pier; The main beam cantilevered with cables was poured until block 29, at which time the right hanging basket and the main beam block 12 simultaneously detached.

According to Fig 18, based on the comparison of working conditions 1 and 2, the detachment of the hanging basket during the construction of the main beam was found to increase the linear elastic stability coefficient of the structure by 0.98%, reflecting a small impact, while the nonlinear stability coefficient decreased by 7.84%.

The comparison of working conditions 2 and 3 reveals that under stiffness values of 0.8 and 1.0 times the stiffness of the main pier, when the hanging basket on the right side detached, the stiffness reduction of the main pier greatly affected the stability of the structure. When the stiffness of the main pier was reduced to 0.8 times the value, the linear elasticity and nonlinear stability coefficients of the structure decreased by 22.22% and 24.47%, respectively. This demonstrates that when the hanging basket detaches while the stiffness of the main pier is simultaneously reduced, the construction stability of the superstructure will be greatly reduced.

Based on the comparison of working conditions 1 and 3, the detachment of the right hanging basket under the reduction of the main pier stiffness to 0.8 times the value was found to significantly reduce the stability of the structure at stage CS71; the linear elastic and nonlinear stability coefficients were respectively reduced by 21.46% and 30.39%.

The analysis of the instability mode revealed that the three working conditions exhibited the same form of instability, i.e., the longitudinal instability of pier #11, as shown in Fig 10c. The combined effect of the reduction in the stiffness of the main pier and the detachment of the construction hanging basket was found to have less impact on the structural instability mode. Therefore, in the stage of maximum cantilever construction with cables, the reduction in the stiffness of the main pier and the detachment of the right hanging basket will individually greatly reduce the construction stability of the structure, and the combined effect of the two factors will cause the stability coefficient to drop even lower. This ultimately increases the risk of structural instability, which is detrimental to construction and should be avoided.

Both multi-factor combinations (i.e., (1) the reduction of the main pier stiffness and asymmetrical construction, and (2) the reduction of the main pier stiffness and the detachment of the hanging basket) were found to significantly reduce the linear elasticity and nonlinear stability of the structure. However, they were found to have little impact on the instability mode. Thus, the combination of unfavorable factors will only have a more adverse impact on the safety and stability of the structure; among the above two combinations of adverse factors, the structure is mainly in the stage of maximum cantilever construction with cables. The combination of the reduction in pier stiffness and the detachment of the hanging basket was found to have the greatest impact on the stability of the structure during construction with the lowest stability coefficient; thus, this combination should be avoided.

## 6 Conclusions

In this study, a stability analysis was conducted on the construction of a partial cable-stayed bridge with multiple towers and high piers. The linear elastic and nonlinear stability coefficients of the main construction stages were calculated and analyzed, as were the instability modes at each stage. Moreover, the sensitivity of the structural stability parameters (the main tower stiffness, main pier stiffness, and main beam stiffness) during the construction stage was examined. Finally, the stability analysis of the structure was conducted based on the combined effects of the most sensitive parameter (the main pier stiffness) and two unfavorable construction factors (the asymmetric construction of the main beam and a detached hanging basket). The specific conclusions of this research are as follows.

1. The stability coefficient of the structure during the entire construction process was found to meet the requirements of the corresponding specifications. The linear elastic stability coefficient at each stage was greater than 4.0, and the nonlinear stability coefficient was greater than 2.5. CS71, the stage of maximum cantilever construction with cables, was found to be the most unfavorable construction stage for the structure. Therefore, during the construction process, the termination of construction at the maximum cantilever stage with cables should be avoided.
2. Based on the stability analysis of the construction stage, from bare tower construction to side-span closure, the instability mode was found to be the longitudinal failure of pier #11; in the secondary mid-span closure stage, the instability mode was found to be the longitudinal failure of pier #12; from mid-span closure to second-phase paving, the instability mode was found to be the overall longitudinal instability of the main pier. Therefore, the main pier has an important impact on the safety and stability of the structure throughout the entire construction process.
3. The parameter sensitivity analysis demonstrated that changes in the stiffness of the main pier have the greatest impact on the safety and stability of the structure, and the stiffness of the main pier is proportional to the structural stability coefficient during the construction stage; the greater the main pier stiffness, the greater the safety and stability coefficients. Therefore, this is a key factor affecting the safety and stability of long-span partial cable-stayed bridge structures with high piers during the construction process.
4. Based on the analysis of multi-factor combinations, the asymmetric construction of the main beam and the detachment of the hanging basket during the construction of the main beam were found to reduce the structural stability during construction. Among the two unfavorable factor combinations, the reduction in the stiffness of the main pier and the detachment of the hanging basket on the right side had the greatest impact on the stability of the structure. While these factors individually have secondary influences, the combination of both factors has a more adverse impact on structural safety and stability. Therefore, construction entities should avoid these two situations when carrying out construction.
5. This article only analyzed the static stability of the structure after the hanging basket detaches during the construction stage. The next research step should be to provide an in-depth discussion of the impact on the structure at the moment when the hanging basket detaches.

## Supporting information

S1 File (XLSX)

## Abbreviations

CS1: bare tower construction
CS22: maximum cantilever construction without cables
CS71: maximum cantilever construction with cables
CS76: side-span closure
CS80: secondary mid-span closure
CS84: mid-span closure
CS87: second-phase paving
p_cr_: critical load of the structure
p_i_: design load under working condition i
λ: stability safety factor of the structure
